# Diseases of the Nose and Throat

**Published:** 1895-03

**Authors:** 


*Diseases of the Nose and Throat.* By F. DE HAVILLAND HALL, M.D. Pp. xii., 524. London: H. K. LEWIS. 1894.

Though several large and complete treatises on diseases of the nose and throat have been published during the last few years, the student and general practitioner will welcome the more handy manual which enables them to make a satisfactory clinical investigation of these regions and to recognise and treat most of the diseases which may arise therein.

Dr. De Havilland Hall is a physician of great experience, and we turned to his work with every expectation of finding a masterly exposition of the subject, but though he has noted every point of practical importance to the practitioner, we confess to a sense of disappointment in that the author seems to have done justice to every laryngologist of eminence but himself. Yet the reader may feel assured that the matter which has been brought into a readable form has been collected and arranged by a skilled hand, and is remarkably accurate and up to date. In this small manual the very numerous references to medical literature have the effect of unduly breaking up the text, while in many instances the references are given to the less important contributions on particular points, and therefore are misleading to those who use them as a guide. The illustrations consist mainly of instruments, and the absence of good figures of the laryngoscopic image in health and disease is a serious defect. Nevertheless, this work will be a valuable addition to the library of every student and practitioner of medicine who feels the need of a practical handbook on laryngology and rhinology.

